# The significance of the social and material environment to place attachment and quality of life: findings from a large population-based health survey

**DOI:** 10.1186/s12955-022-02045-2

**Published:** 2022-09-10

**Authors:** Jan Georg Friesinger, Siri Håvås Haugland, John-Kåre Vederhus

**Affiliations:** 1 Department of Psychosocial Health, University of Agder, Grimstad, Norway; 2 Addiction Unit, Sørlandet Hospital, Kristiansand, Norway

**Keywords:** Quality of life, Wellbeing, Place attachment, Environment, Survey, Norway

## Abstract

**Background:**

There is an international public health interest in sustainable environments that promote human wellbeing. An individual’s bond to places, understood as place attachment (PA), is an important factor for quality of life (QoL). The material environment, such as access to nature (AtN), access to amenities (AtA), or noise, and the social environment, such as social support or loneliness, has the potential to influence PA. The aim of the present study was to explore the relationship between these factors and QoL.

**Methods:**

The study relied on data from 28,047 adults from 30 municipalities in Southern Norway obtained from the Norwegian Counties Public Health Surveys in 2019. Latent regression analyses were used to examine the relationship between the material and social environmental factors and QoL, mediated by PA.

**Results:**

We found a relationship between social and material environmental factors and PA. Higher AtN and AtA scores were related to an increase in PA, whereas higher perception of noise problems was related to decreased PA scores. When social environment factors were added to the model, they were even stronger predictors of PA and, in turn, QoL through mediated effects. We also found a strong positive association between PA and QoL (unstandardized β = 0.88, 95% CI = 0.87–0.90, p < 0.001). The whole model explained 83% of the variance in PA and 65% of the variance in QoL.

**Conclusions:**

Taken together, the findings suggest the relevance of material and social environmental factors for PA and QoL. Therefore, research on public health and QoL should include place-sensitive variables.

**Supplementary Information:**

The online version contains supplementary material available at 10.1186/s12955-022-02045-2.

## Background

In the field of social science and public health, there is increasing interest in how the social and material environment may affect human wellbeing [1–3]. This environmental interest is grounded in the attempt by policymakers to develop sustainable environments that promote quality of life (QoL) across all ages, which is listed as a United Nations (UN) goal and implemented by local governments, including in Norway [4].

The World Health Organization (WHO) offers a standard definition of QoL as “an individual's perception of their position in life in the context of the culture and value systems in which they live and in relation to their goals, expectations, standards, and concerns” [5]. From a place-sensitive research view [6], the “position in life” [5] needs to be seen in an environmental context, including the social and material qualities of places where people are residing and how these matter to their lives. Therefore, the promotion of wellbeing and QoL must involve a broader focus than a solely medical view of human bodies and include the influence of the social and material environments [7–9].

### Conceptual framework

From a place-sensitive perspective, a place refers to a particular somewhere (a geographic location) that can range in scale regarding environmental elements (community, neighbourhood, residence), has a name and a history, wherein people are situated and from which they derive meaning [6, 10, 11]. It is central that people’s experiences with places be relevant to how places influence their wellbeing. Therefore, an individual’s emotional bond to places should be assessed by themselves (e.g., if they feel (dis-)comfort concerning specific place qualities), and one should not solely rely on objective features of the environment. Places matter in various ways for an individual’s ‘position in life’ [5] in terms of QoL and need to be understood in relational ways [12].

#### Place attachment

Place attachment (PA) is commonly defined as a multifaceted concept that characterizes the emotional bonding between people and places [13, 14]. A broadly used concept by Scannell and Gifford [15] considers PA as consisting of person-related (individual or group level) and place-related (e.g., social and material environment) dimensions, in addition to psychological processes (e.g., feelings of affection, belongingness). Instead of understanding the social and material dimensions of place integrated in the PA construct itself, we propose that these factors should be understood as predictors of the emotional bonds people form with places. Two core indicators of the psychological processes are belongingness and feelings of security and safety [14], which draw on the inward/outward relationship of place experiences [11]. As such, the inward aspect of place is understood as belonging in terms of ‘rootedness’ [16], and feeling safe refers to the surroundings (outward aspect) in terms of ‘sense of place’ [17]. Thus, research focuses on particular places where people spend time, reside and to which they feel closely bonded [18]. Although PA can change over time, especially related to dramatic events, PA has been found to be reasonably stable [19]. PA can also be compared to interpersonal attachment, which is known to provide a perceived sense of safety [20, 21].

#### Material environment

Places can have healing powers regarding their material, social, spiritual, and symbolic dimensions [1]; a view that is summarized in the concept ‘therapeutic landscapes’ [22–24]. For example, having access to nature (AtN) can promote health and enhance QoL [25, 26]. Subsequently, happiness is typically reported to be greater in natural environments [27] or in environments with less air pollution and lower noise levels [28]. In addition to nature as a material environment, the built environment has also been found to affect health-related outcomes [29–31]. In many cases, noise is a result of the built environment (e.g., roads, music arenas) and has been identified as a neighbourhood factor that correlates with lower health-related QoL and reduced mental health status of the residents [32, 33]. Another matter is the type of buildings and their materials that influences people’s perceptions of QoL, such as building density, resulting for example in increasing temperature and related discomfort in large cities. The built environment also includes structures entailing amenities, such as services, meeting places, and cultural events. Access to amenities (AtA) provides opportunities for being involved in meaningful activities [34, 35] and is positively associated with well-being [36, 37].

#### Social environment

From a geographic perspective, social capital and social determinants of health can be embedded in a range of spatial scales [3, 38], and social integration, especially at the local level, may have a positive effect on wellbeing and health [39]. Thus, social support and social network in a neighbourhood have been found to increase QoL [40]. Conversely, social isolation and/or loneliness have negative effects on mental health, and can even decrease physical health [41–43], which should be an aim for prevention strategies by policies. Such influence on health is recognized in overarching measures of health, such as QoL [44].

### Objectives

Investigations of both physical constructions (facilities, equipment, organization) and social constructions are important contributions to understanding how places can promote or reduce health as a part of wellbeing [39]. However, very few large-scale, high-quality studies have examined PA and QoL in a latent variable framework (Structural Equation Modeling [SEM]). The main objective of the present study was to examine the relationship between material environment (AtN, AtA, noise), social environment (social support, loneliness), and PA. We also examined whether an association exists between these constructs and QoL, mediated through the PA construct.

We hypothesized that AtA and AtN are positively associated with PA, whereas noise is negatively associated, in line with previous research [14, 45, 46]. We included the predictors of PA in a stepwise/sequential manner and added social variables in the last step. When these variables were added, we expected the explanatory power of the model to increase greatly. In turn, we hypothesized that PA is positively associated with QoL.

## Methods

### Participants and procedures

This study was carried out in the semi-rural county of Southern Norway, which consists of 30 municipalities. Only one municipality had more than 100,000 inhabitants, 11 municipalities had 10,000–45,000 inhabitants, and 18 municipalities had less than 10,000 inhabitants. A randomly selected sample of inhabitants (N = 61,611) of these municipalities aged ≥ 18 years were invited to participate in the Norwegian Counties Public Health Surveys (NCPHS). The selection of participants was based on the Norwegian Population Registry of inhabitants, and contact information (e-mails or telephone numbers) were retrieved from the contact registry from the Agency for Public Management and eGovernment (Difi). A total of 28,047 inhabitants (45.5% response rate) completed the online questionnaire with questions related to local environment, living conditions, health, and well-being.

### Measures

Participant age and sex were provided through the national population registry, and information on education level and relationship status was reported by participants. Economic capability was assessed by a 6-point ordinal item, “Think about the total income in the household: How easy or difficult is it to cope with the family’s daily expenses?” with responses ranging from very difficult to very easy.

#### Material environmental variables

Respondents were asked to think about their local built or natural environment within their municipality. AtN was assessed by two indicators (i.e., access to nature/leisure areas and green areas, such as parks) and AtA by three indicators (i.e., access to service, transport, and culture). Items were scored on 5-point Likert-type scale that ranged from very good to very bad. Scores were reversed and higher scores meant better access. Noise was assessed by two indicators regarding how much respondents were bothered with noise from road traffic and other sources at the place where they reside. A similar 5-point Likert-type scale was used, with responses ranging from “not bothered” to “very much bothered”. Thus, higher scores meant the respondents were more bothered by noise.

#### Social environmental variables

*Social support.* The Oslo Social Support Scale (OSSS) was used. It includes three items assessing social support. First, the question, “How many people are so close to you that you can count on them if you have great personal problems?” has response options as follows: none, 1–2, 3–5, and 5 + . Two additional items are scored on 5-point ordinal scales: “How much interest and concern do people show in what you do?” (response options: from ‘none’ to ‘a lot’), and “How easy is it to get practical help from neighbours if you should need it?” (response options: from ‘very difficult’ to’very easy’) [47]. Thus, higher scores meant higher social support.

*Loneliness.* Subjective feelings regarding the individual’s social situation were assessed with the three-item Loneliness Scale, a short version of the UCLA Loneliness Scale [48]. It contained questions about how often the respondent felt a lack of companionship, being left out, and being socially isolated from others with 5-point response categories ranging from ‘never’ to ‘very often’. with a higher score indicating greater loneliness.

#### Main outcome variables

*Place attachment*. Two indicators were used to assess PA, namely whether respondents felt that they belonged or felt rooted to the place where they reside and felt safe or secure in their local environment regarding this place. We chose our indicators based on the inward/outward relationship of PA described by Seamon [11]. Thus, as suggested above, we propose that the PA construct consists solely of the individual’s affective relationship with place in Scannell’s and Gifford’s [15] definition of PA. The indicators were each rated on scale of 0–10 points, with higher scores representing higher sense of belonging and higher feeling of safety. To the best of our knowledge, these two indicators have not been used previously as sole indicators of PA in a SEM analysis. The analyses provide evidences for convergent validity of the scale, i.e., whether PA is associated with other relevant factors according to hypotheses.

*QoL.* Subjective QoL was measured using three items retrieved from a recommended “minimum” list for measuring QoL in national public health surveys in Norway [49]. The items represented three dimensions of subjective QoL: general happiness, satisfaction with life in general, and sense of meaningfulness in life [50, 51]. The items were each rated on a scale of 0–10 points, with higher scores representing better QoL. The scale was validated in a previous publication [52].

### Statistical analyses

Descriptive statistics were used to show sample characteristics. The hypotheses were tested with latent regression, i.e., the constructs used in the analyses were handled as latent variables in a SEM framework, which means that the questions were modelled as reflective indicators of their respective constructs [53]. With reflective models, causality flows from the latent construct to the observed indicator and the indicators are, in principle, interchangeable, i.e., the interpretation of the model does not change if there are few or many indicators [54]. Thus, constructs can be measured by sampling a few relevant indicators underlying the domain of the construct if one only has a sufficient number of indicators to identify the model. A latent variable analysis includes the error terms as a part of the analysis and, therefore, accounts for and adjusts for measurement error [55].

A sequential analysis strategy was used, and the first model examined the associations of the variables regarding the natural and built (material) environment (AtA, AtN, and noise) with PA and QoL. The second model included variables regarding the social environment with PA and QoL. Mplus version 8.5 and the maximum likelihood (ML) estimator was used for the analyses [56]. Results are reported with the unstandardized beta (β) and 95% confidence interval (CI) to facilitate interpretation of the associations between constructs. In addition, we reported the standardized β to compare the relative strength of the associations between constructs. The significance level was set at p < 0.05. The R^2^ value assessed the percentage of variation in the latent constructs explained by the model. To assess model fit, we used the root mean square error of approximation (RMSEA) and the comparative fit index (CFI); the cut-off value for a good model fit was < 0.06 for RMSEA and > 0.95 for CFI [57]. The full information maximum likelihood (FIML) was used to handle missing values, which is the default procedure in Mplus.

## Results

The mean age of participants was 47 years (SD = 16 years) and slightly more than half (53%) were females (Table 1). Approximately half of participants had a higher education level (at least a bachelor degree) and 8 in 10 lived with a spouse or partner. One in five found it difficult to cover their expenses (Table 1).

**Table 1:** Characteristics of the study sample (N = 28,047)

Characteristic	*Mean (SD)/*n (%)
Age (years)	*47 (16)*
Sex (female)	14,925 (53)
Education level (N = 27,923)	
Primary and secondary school (up to 10 years of education)	3333 (12)
High school (up to 13 years of education)	11,088 (40)
University college or university (≥ bachelor’s degree)	13,502 (48)
Living with a spouse or partner (N = 27,977)	21,893 (78)
Difficult economic capability (N = 26,687)*	5547 (21)

*Proportion reporting it was somewhat difficult, difficult, or very difficult to cope with the family’s daily expenses

**Fig. 1:**
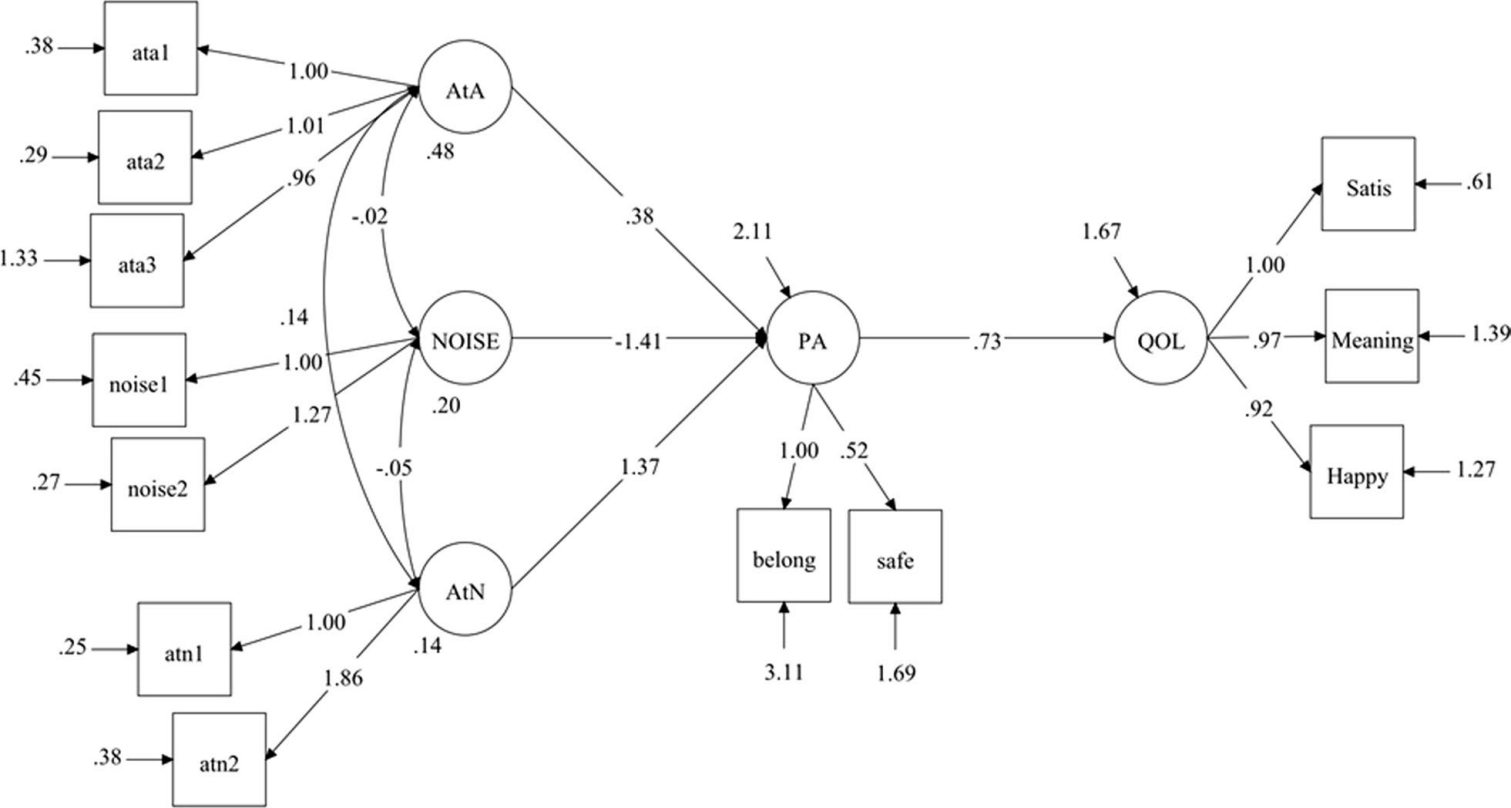
Latent regression model showing associations between material environmental factors as independent variables and quality of life (QoL) mediated by place attachment (PA). The figure shows the measurement and structural model with unstandardized regression coefficients. Access to amenities (AtA), access to nature (AtN)

In the first step of the sequential regression strategy (Fig. 1), we found that AtN (β = 1.37, 95% CI = 1.26/1.48, p < 0.001) and AtA (β = 0.38, 95% CI = 0.33/0.43, p < 0.001) were positively associated with PA, whereas noise was negatively associated with PA (β = –1.41, 95% CI = –1.49 to –1.33, p < 0.001). The standardized β was 0.29, 0.15, and –0.35 for AtN, AtA, and noise, respectively. Thus, noise and AtN were the factors most strongly associated with PA, but in the opposite direction. PA was also positively associated with QoL (β = 0.73, 95% CI = 0.71/0.75, p < 0.001, Fig. 1). The model as a whole explained 34% of the variance in PA and 50% of the variance in QoL, and the model fit was excellent (RMSEA = 0.04 and CFI = 0.98).

**Fig. 2:**
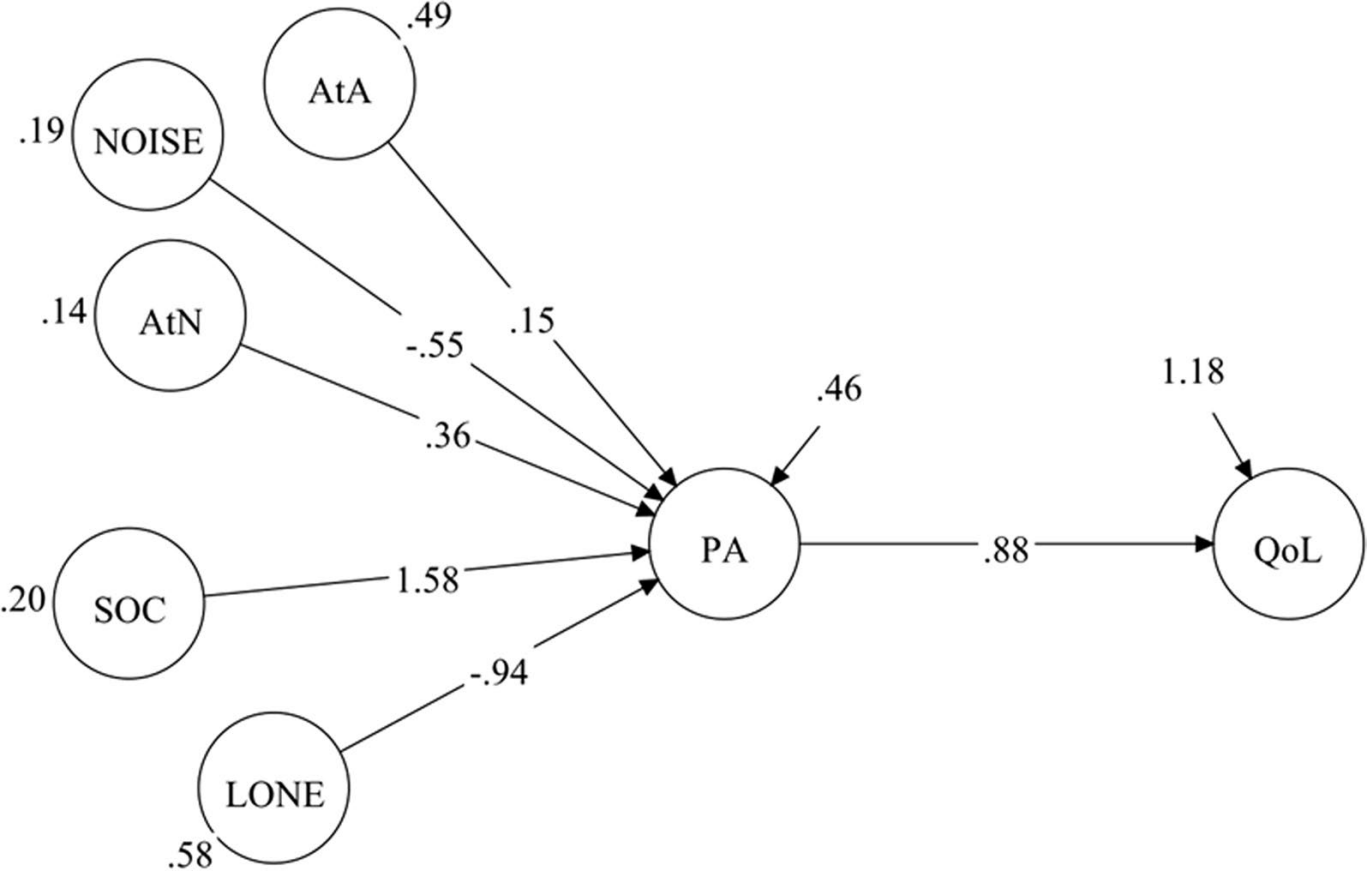
Latent regression model showing associations between material environment and social factors as independent variables and quality of life (QoL) mediated by place attachment (PA). The figure shows the structural model (observed indicators of the latent constructs are not shown) with unstandardized regression coefficients. Access to amenities (AtA), access to nature (AtN), social support (SOC), loneliness (LONE)

When social variables were added to the model, the explained variance of the latent constructs PA and QoL grew substantially to 83% and 65%, respectively. Social support was positively associated with PA (β = 1.58, 95% CI = 1.48/1.68, p < 0.001, Fig. 2), and there was a negative association between loneliness and PA (β = –0.94, 95% CI = − 0.99 to − 0.90, p < 0.001). The regression coefficients between the material environment and PA decreased when the social environment variables were added, indicating that the social environmental variables were stronger explanatory factors for PA. The standardized β values support this; standardized β was 0.43 and –0.43 for social support and loneliness, respectively, but – 0.15, 0.08, and 0.06 for noise, AtN, and AtA, respectively. The positive association between PA and QoL increased (unstandardized β = 0.88, 95% CI = 0.87/0.90, p < 0.001, Fig. 2). When considering the effects mediated from the material and social environmental variables to QoL (the PA β values x the QoL β value), the unstandardized β was − 0.49, 0.32, and 0.13 for noise, AtN, and AtA, respectively. This means, for example, that a one-point higher score for noise was associated with a 0.49 reduction in the QoL score, which is a strong negative association. The effects mediated from the social variables were even stronger, with unstandardized β of 1.40 and − 0.83 for social support and loneliness, respectively. This means, for example, that a one-point higher score on the social support scale was associated with a 1.40 higher QoL score. The fit of the model was excellent (RMSEA = 0.04 and CFI = 0.97).

To control for any potentially confounding effects of the sociodemographic covariates (sex, age, education, relationship, and economic capability), these variables were entered into the model (Additional file 1: Figure S3). When these parameters were added, the fit of the model decreased slightly but was still acceptable (RMSEA = 0.06, CFI = 0.91). Controlling for these covariates had little impact on the main findings reported above. According to the standardized β values (not shown in the figure), economic capability (β = 0.16, p < 0.001), age (β = 0.11, p < 0.001), and living in a relationship (β = 0.04, p < 0.001) were positively associated with PA, whereas education was negatively associated with PA (β = − 0.04, p < 0.001). Gender was not significantly related to PA.

## Discussion

Overall, the findings revealed an association between both social and material environmental factors and PA, which was strongly related to QoL. Higher scores in AtN and AtA were linked to an increase in PA, whereas higher scores in noise were linked to a reduction in PA. Social environmental factors were stronger predictors than material environmental factors in explaining the variation in QoL.

### Environmental factors influence PA and QoL

All three material environmental factors were associated with PA and QoL in the expected directions [14, 45, 46]. AtN was positively associated with PA, confirming our hypothesis and corroborating previous studies on the concept of therapeutic landscapes, in which the bond to nature positively affected QoL [1, 25, 26]. To some extent, we were uncertain that this hypothesis would be supported. On one hand, an Australian study demonstrated that access to green environments does not improve PA [58]. On the other hand, we were aware of the semi-rural property of the present county in our study. The residential density of our setting was low and, even within urban sites, there was still easy access to nature, corresponding with the generally high proportion of 60% of all Norwegian inhabitants having access to recreational areas [59].

As hypothesized, noise from road traffic or other sources was seen as unpleasant/nuisance, similar to air pollution. In line with previous research, our findings indicate that noise can actually reduce PA and, subsequently, QoL [28]. However, we were surprised by the relative strength of the negative association between noise pollution, PA, and QoL. The general concerns raised about noise pollution in Norway since the millennium may partly explain our and others’ findings [60]. The same concern has been seen internationally, and there has been an increased awareness that noise as environmental pollution may be an important source of disease that should be addressed by public health authorities [61]. Cancelling measures as a part of road construction planning is one way to mitigate this problem, as well as constructing main roads away from densely populated areas [62]. Strangely, Félonneau [63] outlined that inhabitants who were bonded to their city experienced noise pollution as less of a problem. In light of our findings, one could speculate as to whether the relatively easy access to nature and noise-free experiences in nature made the present respondents more aware of noise as a phenomenon when they returned back to a noisy neighbourhood. This example outlines once more that planners aiming to promote wellbeing should be aware of the intersection between the built and natural environment [2].

Regarding social environmental factors, social support in the local neighbourhood was found to be a strong predictor of PA and had a positive mediated association with QoL. This finding is in line with studies showing how important social factors are for health and QoL [3, 38, 40, 44]. Our results indicate that social factors were even more potent in influencing PA and QoL than natural and built factors. Therefore, planning sustainable environments [4] should always include a focus on strengthening the ‘social’ environment, not merely the material environment. However, we emphasize the importance of integrating both social and material environments in planning and policy efforts because they interact with one another. Thus, one could intuitively think that AtA would also be relevant as a facilitator of greater social interaction in the local community [34, 35].

### Being emotionally bonded to a place enhances QoL

Perhaps the most surprising of our findings was that the emotional bond between people and places, as well as the model as a whole, was able to explain the variation in QoL at such a high level (65%). Although several authors have found an association between PA and QoL, their results and message were seldom as clear and strong as in the present study [13, 18, 39, 64]. One step up on our 0–10 point scaled PA construct resulted in a 0.88-point increase in QoL, which is a high increase on such a scale. Thus, our findings contribute substantially to the understanding that emotional bonding between people and places is important for people to feel well in life [5].

### Methodological considerations

Among the strengths of this study is its large sample size, with a broad age range, drawn randomly from a general population, and the strong statistical power of our SEM analysis. With latent variable analysis, we are able to assess the strength of relations between latent variables representing constructs, rather than between the measures of the constructs and the analysis controls for the biasing effects of measurement error [65]. The data are based on self-reports, and several of the questions rely on subjective perception (e.g., how much respondents were bothered by noise), rather than objective measures (noise measured in decibels). This subjective approach is based on participant experience, which is closely connected to a similar approach to QoL based on the WHO’s definition highlighting that QoL is about an individual's *perception* of their position in life [5]. The measure of PA was constructive for our analyses and would be valuable to include in future public health studies. Future surveys should include questions about residential mobility, which was not included here. Other research have described sociodemographic factors as being important for PA, with some inconsistent findings within communities with higher residential segregation and social inequality [66]. Importantly, our main findings were not altered by adding sociodemographic variables to our model. A possible reason for this low influence may be that the communities involved with the health survey are characterized by a high degree of welfare and low degree of social inequality [67].

## Conclusions

Our study explained a significant association between social and material environmental factors and QoL, mediated through PA. Social environmental factors social support and loneliness explained more of the variation in PA/QoL than material (built) environmental factors AtN, AtA, and noise. Despite the brevity of our basic indicators for PA and QoL, our model showed its usefulness for public health studies by generating insights that might help policy makers in planning sustainable environments that promote QoL.

## Supplementary Information

**Additional file 1**. **Figure S3 **Latent regression model showing associations between material environment and social factors as independent variables and quality of life (QoL) mediated by place attachment (PA). The model controls for sociodemographic variables. The figure shows the structural model (without the observed indicators of the latent variables) with unstandardized regression coefficients. Abbreviations: access to amenities (AtA), access to nature (AtN), social support (SOC), loneliness (LONE), education (Edu), relationship (Relat), economic capability (INCOME).

## Data Availability

The Controller for this public health survey (NIPH) will deposit the data in a publicly available data repository: https://helsedata.no/en/. For more information, contact the first author.

## Abbreviations

AtA: Access to amenities
AtN: Access to nature
CFI: Comparative fit index
FIML: Full information maximum likelihood
ML: Maximum likelihood
NCPHS: Norwegian Counties Public Health Surveys
OSSS: Oslo Social Support Scale
PA: Place attachment
QoL: Quality of life
RMSEA: Root mean square error of approximation
SEM: Structural equation modeling
UN: United Nations
WHO: World Health Organization
