# Supplementary figures and images for: The significance of the social and material environment to place attachment and quality of life: findings from a large population-based health survey
Source: Health Qual Life Outcomes. 2022 Sep 10;20:135. doi: 10.1186/s12955-022-02045-2 (PMC9463864; doi:10.1186/s12955-022-02045-2)

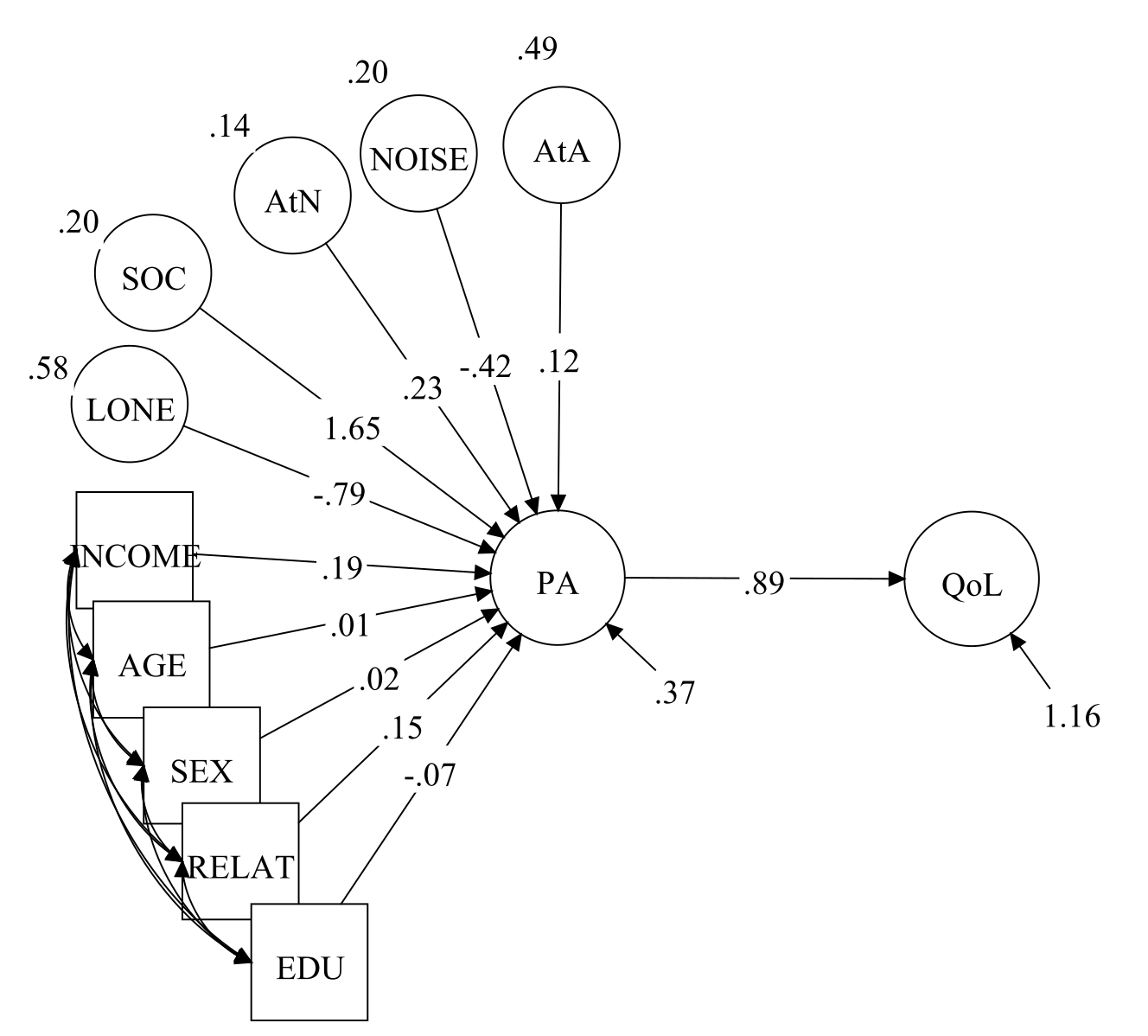

Supplement: Supplementary file 1 — Additional file 1. Figure S3 Latent regression model showing associations between material environment and social factors as independent variables and quality of life (QoL) mediated by place attachment (PA). The model controls for sociodemographic variables. The figure shows the structural model (without the observed indicators of the latent variables) with unstandardized regression coefficients. Abbreviations: access to amenities (AtA), access to nature (AtN), social support (SOC), loneliness (LONE), education (Edu), relationship (Relat), economic capability (INCOME). [file 12955_2022_2045_MOESM1_ESM.jpg]
